# Hemophagocytic lymphohistiocytosis in a patient with COVID-19 treated with tocilizumab: a case report

**DOI:** 10.1186/s13256-020-02503-9

**Published:** 2020-10-15

**Authors:** Birgitte Tholin, Marit Teigen Hauge, Pål Aukrust, Lutz Fehrle, Tor Henrik Tvedt

**Affiliations:** 1 Department of Internal Medicine, Molde Hospital, Molde, Norway; 2 Department of Microbiology, Molde Hospital, Molde, Norway; 3 Research Institute of Internal Medicine, Oslo University Hospital and University of Oslo, Oslo, Norway; 4 Section of Clinical Immunology and Infectious Diseases, Oslo University Hospital, Oslo, Norway; 5 Department of Anesthesiology, Molde Hospital, Molde, Norway; 6 Section of Hematology, Department of Internal Medicine, Haukeland University Hospital, Bergen, Norway

**Keywords:** COVID-19, HLH, Hemophagocytic lymphohistiocytosis, IL-6, Tocilizumab, Case report

## Abstract

**Background:**

The understanding of coronavirus disease 2019 (COVID-19) is rapidly evolving. Although it is primarily a respiratory illness, other manifestations, such as Guillain-Barré syndrome, immune thrombocytopenia, and immune-mediated thrombotic thrombocytopenic purpura, have been described. We present a case of a patient with hemophagocytic lymphohistiocytosis secondary to COVID-19 treated with tocilizumab with a marked biochemical improvement.

**Case presentation:**

In this case report we present a Caucasian patient with COVID-19 who developed a marked elevation of inflammatory parameters with ferritin 36,023 μg/L, but also elevated C-reactive protein 334 mg/L and lactate dehydrogenase 1074 U/L, 1 week after admission to the intensive care unit. He met five of eight criteria for hemophagocytic lymphohistiocytosis, but he lacked the high fever and cytopenia seen in the majority of cases. He was treated with tocilizumab, a monoclonal antibody targeting the interleukin-6 receptor, and over the next days, a rapid decrease in ferritin and C-reactive protein levels was observed. However, his respiratory failure only improved gradually, and he was weaned off the respirator 11 days later.

**Conclusion:**

COVID-19 may induce a hyperinflammatory clinical picture and in some cases develop into hemophagocytic lymphohistiocytosis. In our patient’s case, therapeutic interleukin-6 blockade abrogated signs of hyperinflammation but did not seem to improve pulmonary function. Measurement of ferritin and C-reactive protein, as well as quantification of interleukin-6 on indication, should be performed in patients with severe COVID-19. Specific treatment in such patients must also be contemplated, preferably in randomized controlled trials.

## Introduction

Severe coronavirus disease 2019 (COVID-19) infection, particularly in those with acute respiratory distress syndrome (ARDS), is characterized by marked inflammation and immune activation with elevated levels of inflammatory cytokines, such as interleukin (IL)-6 and tumor necrosis factor [1]. Significantly elevated levels of ferritin as a marker of extensive macrophage activation have also been reported [2]. We describe a case of a patient with a marked systemic immune reaction with features of hemophagocytic lymphohistiocytosis (HLH) who was treated with the IL-6 receptor antagonist tocilizumab.

## Case presentation

A Caucasian man in his 70s was admitted to his local hospital because of fever, diarrhea, and abdominal pain for the previous 10 days. His past medical history was significant for diverticulosis, and he was also being treated for locally advanced prostate cancer with goserelin and for chronic back pain with prednisolone 2.5 mg once daily.

On admission, his blood pressure was 134/85 mmHg, body temperature was 38.7 °C, heart rate was 87 beats/minute, respiratory rate was 18 breaths/minute, and oxygen saturation was 92% without oxygen support. He was slightly overweight with a body mass index (BMI) of 27 kg/m^2^, but the findings of his physical examination were otherwise normal. His laboratory test values, presented in Table 1, showed elevated C-reactive protein (CRP), thrombocytopenia, and moderately elevated ferritin. Arterial blood gas analysis revealed respiratory alkalosis with hypoxemia and normal lactate. A radiographic examination showed scattered consolidations in the right lung.

**Table 1:** An overview of relevant laboratory parameters during the clinical course

Parameter	Reference range	Admission Day 0	ICU Day 2	Day 5	Day 6	Day 7	Day 8	Extubation Day 18
Hemoglobin (g/dl)	13.7–16.5	12.9	11.7	11.3	11.8	11.1	11.3	8.8
Neutrophils (10^9^/L)	1.5–5.7	6.6	–	–	20.3	31.3	30.5	8.0
Lymphocytes (10^9^/L)	1.3–3.4	1.6	–	–	–	–	–	–
Platelet count (10^9^/L)	145–390	95	107	179	197	190	177	222
Creatinine (mg/dl)	60–105	115	91	297	339	233	155	156
CRP (mg/L)	0–4	167	224	315	348	276	63	12
Ferritin (μg/L)	23–431	544	635	6399	36,023	75,920	20,730	625
Bilirubin (μmol/L)	5–25	8	7	32	39	47	60	10
LD (U/L)	115–255	522	583	–	1074	1559	973	438
Fibrinogen (g/L)	2–4	–	–	–	6.5	5.9	–	–
Triglyceride (mmol/L)	0.45–2.60	–	–	–	5.27	5.71	–	–

*Abbreviations: CRP* C-reactive protein, *ICU* Intensive care unit, *LDH* Lactate dehydrogenase

The patient had a positive test result for severe acute respiratory syndrome coronavirus 2 (SARS-CoV-2) based on real-time reverse transcriptase polymerase chain reaction of a nasopharyngeal specimen. Empirical treatment with cefotaxime and ciprofloxacin was prescribed for suspected bacterial superinfection as well as an antiviral regime of lopinavir-ritonavir and hydroxychloroquine according to local guidelines at the time.

Twelve hours later, the patient’s condition deteriorated, with rapidly progressing respiratory failure, oxygen saturation at 86% on 12-L oxygen, and a respiratory frequency of 40 breaths/minute. This prompted transfer to the intensive care unit (ICU) and intubation. A new chest x-ray revealed extensive bilateral coalescent opacities qualifying as severe ARDS (ratio of arterial oxygen partial pressure to fractional inspired oxygen [FiO_2_], ≤ 100 mmHg).

During the first 36 hours in the ICU, the patient was in unstable cardiopulmonary condition. He required high FiO_2_ and norepinephrine in moderate doses, and he developed supraventricular tachyarrhythmia that was treated with repeated electrical and pharmacological cardioversion. By day 7, he had accumulated significant amounts of fluid (positive fluid balance of 8 L). His creatinine levels were rising, and he responded poorly to diuretics. Continuous venovenous hemodiafiltration was initiated to ensure a negative fluid balance.

The antiviral drug regimen was discontinued after 3 days due to a national agreement that all SARS-CoV-2 viral drug treatments should be administered through randomized controlled studies. Due to rising CRP and leukocyte counts, the antibiotics were changed to meropenem.

One week after admission, the patient achieved circulatory stability and exhibited a slowly decreasing oxygen demand, but his ferritin had risen markedly to 36,023 μg/L. This was accompanied by occasional fever and marked increases in CRP (334 mg/L), lactate dehydrogenase (LDH) (1074 U/L), neutrophil count (20.3 × 10^9^/L), and triglycerides (5.27 mmol/L). His triglycerides were analyzed during parenteral nutrition and must be interpreted with caution.

This raised concern that the patient had developed HLH secondary to SARS-CoV-2. His soluble IL-2 receptor level was considerably elevated at 6809 U/ml (> 623 U/ml indicates immune activation and T-cell activation in particular), and a bone marrow smear demonstrated hemophagocytosis. Flow cytometry of peripheral blood showed a significant decrease in circulating CD4^+^ and CD8^+^ T cells (161/μl and 32/μl, respectively) but an expanded population of clonal B cells that expressed kappa, CD5, CD19, CD20 (weakly), CD43, CD45, and CD200. Due to the absence of lymphocytosis, and after a review of laboratory records, this was classified as monoclonal B-cell lymphocytosis (MBL) and not as chronic lymphocytic leukemia (CLL), which requires > 5000 cells/μl. A trephine biopsy confirmed MBL but no other lymphoproliferative disorders.

The patient fulfilled five of eight HLH-2004 diagnostic criteria, and his H-score estimated the probability of HLH to be 96–98% [3, 4]. A decision was made to give the patient tocilizumab 800 mg intravenously, a monoclonal antibody against the IL-6 receptor that is used when cytokine release syndrome (CRS) is seen following the infusion of chimeric antigen receptor T cells.

**Fig. 1:**
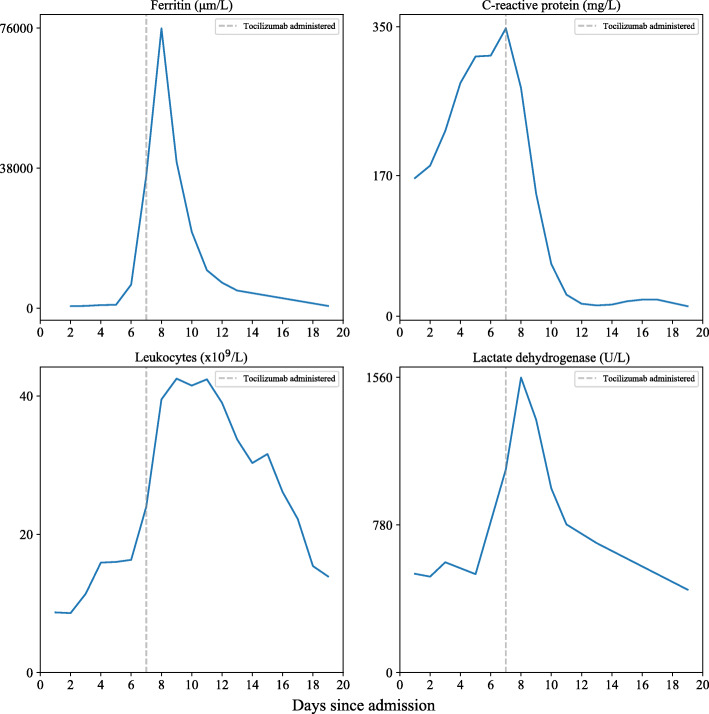
Plot of relevant laboratory values: ferritin, leukocytes, C-reactive protein, and lactate dehydrogenase. The horizontal axis represents days from admission, and the vertical axis represents the laboratory values in proportion to the peak value

The following day, the patient’s CRP declined rapidly, followed by a significant but slow decline in ferritin and LDH levels (Table 1, Fig. 1). After the administration of tocilizumab, no fever was observed. Three days later, the patient also received one dose of intravenous immunoglobulins pending the result of protein electrophoresis, which was normal. His clinical state improved 5–7 days later, and he was successfully extubated.

After the patient’s improvement, we analyzed serum levels of selected cytokines on the same day as tocilizumab was given (samples taken before administration) by enzyme-linked immunosorbent assay. Interestingly, his IL-6 levels were markedly elevated at 84 pg/ml (< 5 pg/ml), whereas TNF and IL-10 were moderately elevated at 55 pg/ml (< 20 pg/ml) and 38 pg/ml (< 5 pg/ml), respectively. His IL-8 (66 pg/ml) and IL-1β (< 5 pg/ml) were within the normal range of the laboratory that did the tests (Sahlgrenska University Hospital, Gothenburg, Sweden).

## Discussion and conclusions

We report a case of a patient with marked immune activation accompanied by ARDS with a biochemical pattern consistent with secondary HLH who responded biochemically after one dose of the IL-6 receptor antagonist tocilizumab. HLH is characterized by hyperinflammation; fever; multiorgan involvement; and, in severe cases, marked hemophagocytosis with cytopenia. Although primary HLH is caused by genetic defects that impair natural killer (NK) or T-cell function, secondary HLH is caused by various disorders, most commonly malignancies, infections, and autoimmune disorders. HLH is diagnosed either by detection of HLH-associated mutations (diagnostic for primary HLH) or by verification of a minimum of five of the following eight criteria: fever > 38.5 °C, splenomegaly, cytopenia, fasting hypertriglyceridemia (> 265 mg/dl) and/or hypofibrinogenemia (< 150 mg/dl), hemophagocytosis, low or absent NK cell activity, ferritin > 500 ng/ml, and soluble IL-2receptor elevated 2 SD above age-adjustedlaboratory-specific norms. Macrophage activation syndrome (MAS) shares some feature of HLH, such as strong macrophage activation and excessive hyperferritinemia. MAS is associated with autoimmune diseases, especially juvenile idiopathic arthritis, systemic lupus erythematosus, Kawasaki disease, and juvenile dermatomyositis [5].

Both primary and secondary HLH can be triggered by viral infections, Epstein-Barr virus infection in particular [6]. There are several reports of cytokine “storm” or hyperinflammation in patients with COVID-19 with elevated levels of several inflammatory cytokines, but in most of these cases, there are no other signs of HLH, and the ferritin level is seldom > 35,000 μg/L as in our patient’s case [1, 7]. The mechanisms of this SARS-CoV-2–triggered “immune storm” that can develop into HLH are currently unclear, but several not mutually exclusive mechanisms could be operating. *In vitro* studies have suggested that SARS-CoV-2 could activate NLRP3 inflammasomes that are potent activators of macrophages, with a marked release of IL-1β as a consequence, which subsequently contributes to IL-6 release [8]. Furthermore, molecular docking analysis predicts spontaneous interaction between the spike glycoprotein of SARS-CoV-2 and Toll-like receptors 5 (TLR5). TLR5 is expressed on monocytes and significantly contributes to IL-6 release thorough activation of nuclear factor-κB activation [9]. Moreover, through downregulation of the angiotensin (AT)-converting enzyme 2, the putative SARS-CoV-2 receptor, the virus could induce a downregulation of the antiproliferative and anti-inflammatoryAT-1–7 pathway [10].

Infection with SARS-CoV-2 induces a great variety of clinical symptoms, ranging from asymptomatic to mild or critical. Although severe illness can occur in otherwise healthy individuals of all ages, it predominantly occurs in individuals older than 65 years of age [11]. One factor associated with a severe course of SARS-CoV-2 infection is impaired or delayed T-cell response [12]. Interestingly, we were able to verify the presence of clonal B cells with the CLL immunophenotype in our patient. Due to the low number of circulating lymphocytes, the condition was characterized as MBL. Clonal lymphocytosis occurs in < 0.5% of the population under the age of 40 but increases significantly with age and can be demonstrated in 10–20% of individuals over 70 years old, increasing even more with age [13]. MBL increases susceptibility to viral infections through several mechanisms, including hypogammaglobulinemia, the accumulation of incompetent lymphocytes with an impaired response to immunological stimuli, and a reduced cytotoxic capacity of CD8^+^ cells due to reduced granzyme levels and the nonpolarized degranulation of vesicles [14]. Furthermore, it is well known that the immune dysfunction in some patients with CLL can result in HLH [15].

Other possible influencing factors in our patient were prolonged obesity, treatment with low-dose steroid, and medical castration with goserelin. Goserelin has not been reported to have immunological side effects but is associated with a weight increase in approximately 5% of patients. This, in addition to steroids, could have contributed to his raised BMI which is a recognized risk factor for developing severe COVID-19. Whether these factors influenced the clinical course in our patient is speculative.

Secondary HLH, MAS, and CRS constitute a heterogeneous group of conditions that are all characterized by macrophage activation and excessive cytokine production, and IL-6 seems to be involved in various degrees in all these conditions. Such mechanisms could also be operating in severe COVID-19 infection. Thus, there are some reports of beneficial effects of tocilizumab in severe COVID-19, and randomized studies are ongoing [16]. Our patient had markedly elevated IL-6 levels and a profound and rapid decline in CRP as a biomarker of IL-6 activity, further supporting a role for IL-6–driven inflammation in severe COVID-19 infection. Notably, whereas there was a rapid decline in CRP, there was an incidental increase in ferritin following administration of tocilizumab before a significant decline, suggesting that the effect of tocilizumab on macrophage activation involves secondary effects of IL-6. An early and significant rise in IL-6 levels during SARS-CoV-2 infections is associated with a poor outcome. One should consider screening with ferritin and CRP in cases of severe COVID-19 with apparent hyperinflammation, particularly because CRP is known to be an indirect marker of IL-6 [17]. With significantly elevated levels, the diagnosis could be further supported by quantitating IL-6.

## Data Availability

NA.

## References

[1] Mehta P, McAuley DF, Brown M, *et al.* COVID-19: consider cytokine storm syndromes and immunosuppression. Lancet. 2020;395:1033–4. 10.1016/S0140-6736(20)30628-0 PMC7270045 32192578

[2] Lambotte O, Cacoub P, Costedoat N, Le Moel G, Amoura Z, Piette JC. High ferritin and low glycosylated ferritin may also be a marker of excessive macrophage activation. J Rheumatol. 2003;30(5):1027–8. 12734900

[3] Jordan MB, Allen CE, Weitzman S, Filipovich AH, McClain KL. How I treat hemophagocytic lymphohistiocytosis. Blood. 2011;118(15):4041–52. 10.1182/blood-2011-03-278127 PMC3204727 21828139

[4] Debaugnies F, Mahadeb B, Ferster A, *et al.* Performances of the H-score for diagnosis of hemophagocytic lymphohistiocytosis in adult and pediatric patients. Am J Clin Pathol. 2016;145(6):862–70. 10.1093/ajcp/aqw076 27298397

[5] Lin CI, Yu HH, Lee JH, *et al.* Clinical analysis of macrophage activation syndrome in pediatric patients with autoimmune diseases. Clin Rheumatol. 2012;31(8):1223–30. 10.1007/s10067-012-1998-0 22615046

[6] Filipovich A, McClain K, Grom A. Histiocytic disorders: recent insights into pathophysiology and practical guidelines. Biol Blood Marrow Transplant. 2010;16(1 Suppl):S82–9. 10.1016/j.bbmt.2009.11.014 19932759

[7] Zhang C, Wu Z, Li JW, Zhao H, Wang GQ. Cytokine release syndrome in severe COVID-19: interleukin-6 receptor antagonist tocilizumab may be the key to reduce mortality. Int J Antimicrob Agents. 2020;55(5):105954. 10.1016/j.ijantimicag.2020.105954 PMC7118634 32234467

[8] Zhao C, Zhao W. NLRP3 inflammasome—a key player in antiviral responses. Front Immunol. 2020;11:211. 10.3389/fimmu.2020.00211 PMC7040071 32133002

[9] Bhattacharya M, Sharma AR, Patra P, *et al.* Development of epitope-based peptide vaccine against novel coronavirus 2019 (SARS-COV-2): immunoinformatics approach. J Med Virol. 2020;92(6):618–31. 10.1002/jmv.25736 PMC7228377 32108359

[10] Tignanelli CJ, Ingraham NE, Sparks MA, *et al.* Antihypertensive drugs and risk of COVID-19? Lancet Respir Med. 2020;8(5):e30–1. 10.1016/S2213-2600(20)30153-3 PMC7194709 32222166

[11] Epidemiology Working Group for NCIP Epidemic Response, Chinese Center for Disease Control and Prevention. The epidemiological characteristics of an outbreak of 2019 novel coronavirus diseases (COVID-19) in China [in Chinese]. Zhonghua Liu Xing Bing Xue Za Zhi. 2020;41(2):145–51. 10.3760/cma.j.issn.0254-6450.2020.02.003 32064853

[12] Zheng HY, Zhang M, Yang CX, *et al.* Elevated exhaustion levels and reduced functional diversity of T cells in peripheral blood may predict severe progression in COVID-19 patients. Cell Mol Immunol. 2020;17(5):541–3. 10.1038/s41423-020-0401-3. PMC7091621 32203186

[13] Strati P, Shanafelt TD. Monoclonal B-cell lymphocytosis and early-stage chronic lymphocytic leukemia: diagnosis, natural history, and risk stratification. Blood. 2015;126(4):454–62. 10.1182/blood-2015-02-585059 PMC4624440 26065657

[14] Lanasa MC, Weinberg JB. Immunologic aspects of monoclonal B-cell lymphocytosis. Immunol Res. 2011;49(1–3):269–80. 10.1007/s12026-010-8188-4 PMC4102133 21161696

[15] Bailey C, Dearden C, Ardeshna K. Haemophagocytic lymphohistiocytosis as a consequence of untreated B-cell chronic lymphocytic leukaemia. BMJ Case Rep. 2017;2017:bcr2016219057. 10.1136/bcr-2016-219057 PMC5534911 28446487

[16] Xu X, Han M, Li T, et al. Effective treatment of severe COVID-19 patients with tocilizumab. Proc Natl Acad Sci U S A. 2020;117(20):10970-10975. 10.1073/pnas.2005615117. PMC7245089 32350134

[17] Del Giudice M, Gangestad SW. Rethinking IL-6 and CRP: Why they are more than inflammatory biomarkers, and why it matters. Brain Behav Immun. 2018;70:61–75. 10.1016/j.bbi.2018.02.013 29499302

